# Multi-UAV Path Planning Algorithm Based on BINN-HHO

**DOI:** 10.3390/s22249786

**Published:** 2022-12-13

**Authors:** Sen Li, Ran Zhang, Yuanming Ding, Xutong Qin, Yajun Han, Huiting Zhang

**Affiliations:** 1 School of Information Engineering, Dalian University, Dalian 116622, China; 2 Communication and Network Laboratory, Dalian University, Dalian 116622, China

**Keywords:** multiple unmanned aerial vehicles, Harris hawks optimization, bioinspired neural network, energy cycle decline mechanism, dynamic obstacle avoidance

## Abstract

Multi-UAV (multiple unmanned aerial vehicles) flying in three-dimensional (3D) mountain environments suffer from low stability, long-planned path, and low dynamic obstacle avoidance efficiency. Spurred by these constraints, this paper proposes a multi-UAV path planning algorithm that consists of a bioinspired neural network and improved Harris hawks optimization with a periodic energy decline regulation mechanism (BINN-HHO) to solve the multi-UAV path planning problem in a 3D space. Specifically, in the procession of global path planning, an energy cycle decline mechanism is introduced into HHO and embed it into the energy function, which balances the algorithm’s multi-round dynamic iteration between global exploration and local search. Additionally, when the onboard sensors detect a dynamic obstacle during the flight, the improved BINN algorithm conducts a local path replanning for dynamic obstacle avoidance. Once the dynamic obstacles in the sensor detection area disappear, the local path planning is completed, and the UAV returns to the trajectory determined by the global planning. The simulation results show that the proposed Harris hawks algorithm has apparent superiorities in path planning and dynamic obstacle avoidance efficiency compared with the basic Harris hawks optimization, particle swarm optimization (PSO), and the sparrow search algorithm (SSA).

## 1. Introduction

The development of network information technology has promoted the emergence and rapid development of UAV clusters [1]. Multi-UAV cooperative combat has become a significant development trend in future air combat. Compared with a single UAV, multi-UAV afford a higher combat effectiveness and stronger combat ability. Path planning [2] technology provides path guidance for the UAVs, which is one of the key techniques to achieve cooperative combat with UAVs [3].

In the actual flight of UAVs, there are still some problems, such as a slow calculation speed, long flight path, unreasonable path planning, or inability to effectively reduce fuel consumption. Therefore, how to improve the safety and effectiveness of flight has become a practical problem to be solved.

Currently, multi-UAV path planning consists of global path planning [4] and local path planning [5]. Global path planning means planning paths based on all information in a given environment, with the literature suggesting several swarm intelligence algorithms [6], such as the red deer algorithm (RDA) [7], pigeon-inspired optimization (PIO) [8], whale optimization algorithm (WOA) [9], ant colony optimization (ACO) [10], and sparrow search algorithm (SSA) [11]. Local path planning refers to selecting the UAVs’ next flight direction according to the current environmental constraints and dynamic obstacles, effectively improving the UAVs’ dynamic obstacle avoidance capability.

Obstacle avoidance [12] is key to realizing autonomous and intelligent UAV flights. A perfect autonomous obstacle avoidance system should significantly reduce the damage accident rate caused by UAV operation errors. The primary purpose of UAV dynamic obstacle avoidance is to make the UAV sense the position and speed of its surrounding obstacles in real time. Compared with static obstacle avoidance, the core difference is that the UAV must predict the obstacle location in real time and change its flight direction in advance to achieve obstacles avoidance.

Therefore, aiming at the dynamic obstacle avoidance problem during the flight of multi-UAVs, this paper introduces an energy cycle decline mechanism to improve the traditional HHO algorithm, and combines it with a BINN. The UAVs’ path planning switches between the improved HHO algorithm and the BINN according to the sensor’s judgment of obstacles, so that the UAVs can effectively avoid static and dynamic obstacles, improving the computing speed and convergence ability.

The rest of this paper is organized as follows. Section 2 shows related works. The path planning modeling and constraints are then illustrated in Section 3. The overview of HHO is given in Section 4. Section 5 introduces the specific content of improved BINN-HHO algorithms for multi-UAV path planning. Section 6 carries out simulation experiments and analyzes the results in four cases. Finally, Section 7 summarizes this research and introduces future research directions.

## 2. Related Works

In recent years, researchers have conducted a lot of studies in the field of UAV path planning, both in global and local path planning.

For global path planning, Ji et al. [13] proposed a double-dynamic learning PSO algorithm to optimize multiple terrain problems. Huang et al. [14] combined reinforcement learning and PSO (RMPSO) for rapid path planning and practical obstacle avoidance for autonomous underwater vehicles (AUVs). Liu et al. [15] adopted an adaptive weight of inertia to balance the algorithm’s exploration power and convergence rate. Ma et al. [16] suggested a mixed solution based on an artificial bee colony (ABC) algorithm’s life cycle to generate dynamically varying populations and ensure an applicable balance between exploration and exploitation. Yan et al. [17] introduced a WOA based on a forward-looking sonar to achieve the 3D path planning of UAVs. This work was also established for two-dimensional (2D) optimal path planning [18]. Wang et al. [19] proposed an improved ACO algorithm with dynamic adaptive parameters to solve the path planning problem. At the same time, Yan [20] suggested an optimization strategy of UAV mission planning based on a Gauss perturbation ACO algorithm to improve the effectiveness of UAV mission planning. Yu et al. [21] developed a sparrow particle swarm (SPSA) algorithm to optimize UAV path planning, which selected an appropriate model for trajectory initialization, changed the update of a finder’s position, strengthened the influence of the starting and ending lines on the path search, and reduced blind search significantly. Although global path planning is simple and has low calculation requirements, it cannot solve the dynamic obstacle avoidance problem effectively.

For local path planning, Qie et al. [22] proposed an artificial intelligence (AI) approach based on a multiagent depth deterministic strategy gradient (MADDPG) to deal with dynamic environments effectively. Liu et al. [23] proposed a dynamic path planning method for unknown environments that combined a little prior knowledge and real-time survey results to improve the autonomous navigation ability of UAVs in a complex environment. Yao et al. [24] suggested a rolling-optimization feedback algorithm named model predictive control (MPC) to avoid sudden and mobile threats dynamically. Feng et al. [25] proposed a modified artificial potential field to predict the obstacles’ positions and solve local oscillations or avoid local minima simultaneously. Additionally, Wang et al. [26] predicted the late motion state of the target according to the target position and combined the radar feedback data and state for dynamic path planning. These schemes improved UAV intelligence and allowed long-term tracking. However, due to the local planning calculation complexity, this method imposed high computational requirements on the UAV that made it difficult to achieve global optimization.

Recently, since the HHO algorithm has the advantages of few parameters and simple calculation, it has often been used in UAV path planning. Heidari et al. proposed the HHO algorithm [27] in 2019 inspired by the Harris hawks’ predation behavior, which included search and development phases. HHO has fewer parameters and a stronger global exploration ability compared with other swarm intelligence algorithms. However, when solving complex optimization problems, the HHO algorithm suffers from a low optimization accuracy and easily falls into a local optimization. Hence, Qu et al. [28] proposed an improved HHO that utilized information exchange to optimize HHO’s continuous function and applied it to engineering problems. Moreover, Krishna et al. [29] suggested the hybrid Harris hawks algorithm using the pattern search algorithm (hHHO-PS) to enhance the global search process of the current HHO and extend HHO’s local search space constraints. Zhang et al. [30] introduced an evolutionary algorithm based on an improved ABC algorithm that exploited a reverse learning Harris hawks (HABC) scheme to acquire a high convergence accuracy and fast convergence speed. Nandi et al. [31] combined HHO with the grey wolf optimizer (hHHO-GWO) to solve nonlinear, nonconvex, and highly constrained engineering design problems. Liu [32] developed an improved HHO variant to overcome the shortcomings of blindness in the global exploration phase. Although the above-improved HHO algorithm have achieved good results in different fields, there is little research in the field of multi-UAV path planning.

In order to overcome the existing problems, this paper proposes an effective path planning algorithm that consists of a bioinspired neural network [33] and improved Harris hawks optimization algorithm with a periodic energy decline regulation mechanism (BINN-HHO). The developed algorithm uses numerical coding to simulate the terrain, with smooth surfaces obtained through interpolation. When the UAV sensor does not detect a dynamic obstacle, a periodic energy decline regulation mechanism [34] is introduced into the HHO and embedded into the energy function to conduct global multi-UAV path planning. When the sensor detects a dynamic obstacle during flight, it starts from its current position and employs the improved BINN method to replan the local path. When the dynamic obstacles in the sensor detection area disappear, the UAVs complete the local path planning and return to the predetermined trajectory of the global path planning. Compared with the traditional path planning algorithm, the proposed algorithm in this paper realizes a multiround dynamic iterative balance between global exploration and local exploitation of the HHO algorithm and a real-time switching between static obstacle avoidance and dynamic obstacle avoidance during UAV flight, which improve the shortcomings of traditional methods such as a poor dynamic obstacle avoidance effect and the ease of falling into a local optimization, and a safer and faster UAV flight path can be generated in an environment with dynamic obstacles.

## 3. Modeling and Constraints

This paper studies the algorithm of the UAVs’ flight path, in which a UAV is considered as a node without specifically analyzing the control structure inside the UAV. Additionally, since conventional fixed-wing UAVs do not have vertical takeoff ability and cannot hover, this paper assumes that UAVs are multirotor UAVs that can adjust their flight speed and height according to nodes and terrain.

Before path planning, we need to model the environment and barriers and set environmental conditions. This section first introduces the establishment of the environmental model, then sets the dynamic obstacle avoidance constraints, and finally describes the path cost and path constraints.

### 3.1. Environmental Modeling

**Figure 1:**
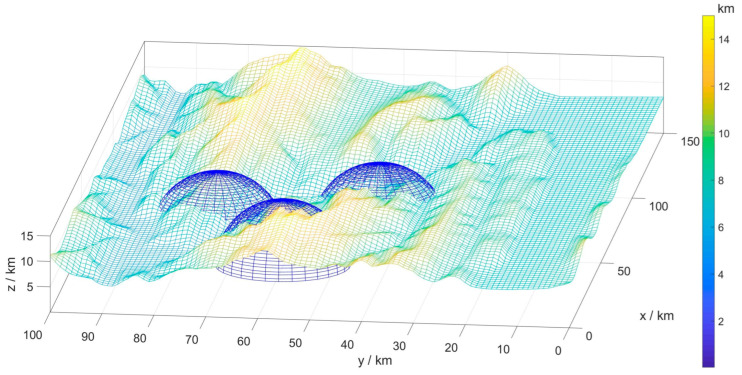
Three-dimensional digital map.

In order to simulate the real environment of UAV flight, a real battlefield environment with dynamic and static obstacles was established. In this paper, the terrain was simulated by numerical coding, and the peaks and valleys were described as matrices, where the matrix values represented the terrain elevation at the current coordinate position. The terrain was finally smoothed and simulated by interpolation (Figure 1).

This study considered the detection range of an enemy radar as the threat area, calculated using Equation (1). The blue hemispheres in Figure 1 simulate and model the threat areas.

(1) $$W_{i}(x,y,z)=\{\begin{array}{c} \sum_{i}{\left(x-x_{i}\right)}^{2}+{\left(y-y_{i}\right)}^{2}+{\left(z-z_{i}\right)}^{2}=R_{i}^{2}\\ z\geq 0 \end{array}$$

where *W_i_* (*x, y, z*) denotes threat area *i*, *(x_i_, y_i_, z_i_)* is the location of the radar, and *R_i_* is the radar’s detection radius.

When the UAV detects dynamic obstacles during its flight, it starts a local path replanning, which requires local modeling according to the current environment.

**Figure 2:**
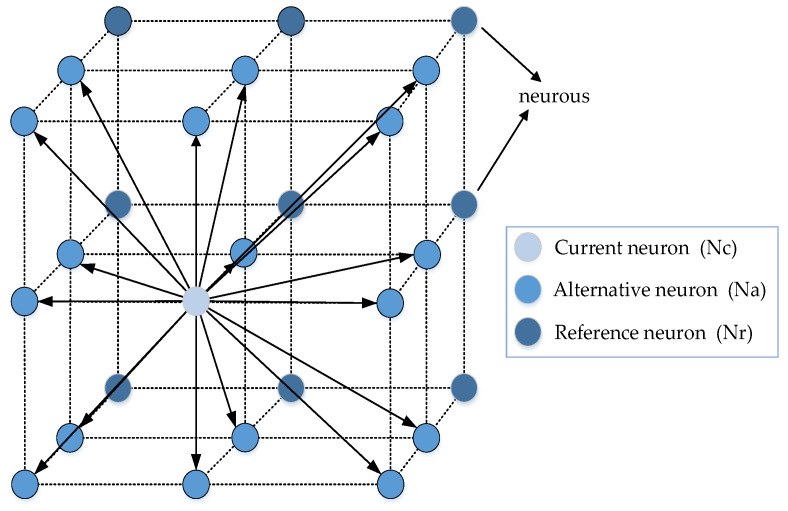
Local space model.

Specifically, a local space was defined, and a 3 × 3 × 3 grid diagram comprising three types of neurons was established, connected as illustrated in Figure 2. When the UAV detects dynamic obstacles, there are 17 optional flight directions represented by neuron Na. At the same time, the reference neuron Nr information is also examined. When the neuron Nr behind Na is occupied by an obstacle, the neuronal activity information of Na decreases, and the reference neuron Nr can be used as a buffer for UAV dynamic obstacle avoidance to improve the success rate.

When the UAV starts to avoid local obstacles, the motion information of the forwarding obstacles is detected by the UAV’s onboard sensors, expressed as the state information of each neuron. The state information of the replacement neuron Na and the reference neuron Nr are defined in Equations (2) and (3), respectively.

(2) $$\mathrm{Na}=\{\begin{array}{c} 2E,\mathrm{target}\;\;\mathrm{location}\\ E,\mathrm{movable}\;\;\mathrm{position}\\ -2E,\mathrm{obstacle}\;\;\mathrm{position}\\ 0,\mathrm{UAV}\;\;\mathrm{position} \end{array}$$

(3) $$\mathrm{Nr}=\{\begin{array}{c} -2E,\mathrm{obstacle}\;\;\mathrm{position}\\ 2E,\mathrm{target}\;\;\mathrm{location}\\ 0,\;\;\mathrm{otherwise} \end{array}$$

### 3.2. Cooperative Obstacle Avoidance Constraints

During its flight, the UAV needs to avoid the detection of enemy radars, and at the same time prevent collisions with other UAVs. Therefore, this paper considered the reconnaissance area of the enemy radar and the safe distance between the UAVs as a threat zone for UAVs’ flight, and we set cooperative obstacle avoidance constraints.

**Figure 3:**
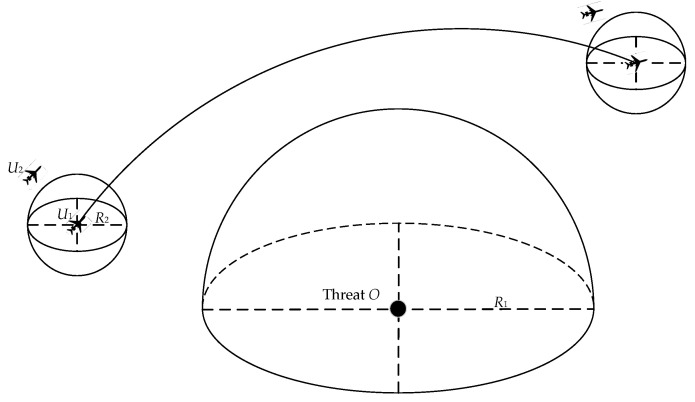
Cooperative obstacle avoidance model.

The threat areas, including radar detection areas and the safe distance between UAVs, are illustrated in Figure 3, defined by the semisphere formed at the threat source *O* (*x_o_*, *y_o_*, *z_o_*) with an action radius of *R*_1_. The UAVs need to avoid this threat area during flight successfully.

Hence, during the flight path from key node *X* to key node *X +* 1, the distance between threat source *O* and any point *T* (*x_t_*, *y_t_*, *z_t_*) should meet the following requirements:

(4) $$R_{1}<\sqrt{{\left(x_{t}-x_{o}\right)}^{2}+{\left(y_{t}-y_{o}\right)}^{2}+{\left(z_{t}-z_{o}\right)}^{2}}$$

Multi-UAV must keep a safe distance to avoid collisions between two or more UAVs flying cooperatively. Therefore, a UAV was considered as the center and the minimum distance *R*_2_ between two UAVs as the radius to construct a sphere. The distance between two adjacent *U*_1_ (*x*_1_, *y*_1_, *z*_1_) and *U*_2_ (*x*_2_, *y*_2_, *z*_2_) must conform to the following formula:

(5) $$R_{2}<\sqrt{{\left(x_{1}-x_{2}\right)}^{2}+{\left(y_{1}-y_{2}\right)}^{2}+{\left(z_{1}-z_{2}\right)}^{2}}$$

### 3.3. Path Cost Function

In order to evaluate the quality of the final mapped path, a path cost function was established. The specific formula was as follows

(6) $$F=\sum_{i=1}^{n}\left(\omega_{1}l_{i}+\omega_{2}h_{i}+\omega_{3}f_{i}\right)$$

where *n* means the path is divided into *n* segments, *L_i_* (*i* = 1, 2, …, *n*) means the path length of section *i*, *h_i_* is the average flight height of section *i*, and *f_i_* means the composite threat index of section *i*. *ω*_1_, *ω*_2_, and *ω*_3_ represent the weight coefficients corresponding to path length, average flight height, and composite threat index, respectively. The *f_i_* was calculated using Equation (7).

(7) $$f_{i}=\frac{\sum_{j=1}^{m}Q_{ij}}{{\left(D_{ij}\right)}^{4}}$$

where *m* is the total number of threat points, *Q_ij_* (*j* = 1, 2, …, *m*) representing the threat index of the path of section *i* to the threat point *j* that the control center can collect, and *D_ij_* means the distance between the threat point *j* and UAV in section *i*.

In the individual fitness calculation, it is indispensable to normalize the substitution value of each part in the path cost function to avoid calculation errors caused by the order of magnitude difference of each part replacement value.

### 3.4. Path Constraints

Considering the actual flight environment and UAV performance limitations, the path of UAV must meet some specific constraints [35]. Therefore, it is necessary to constrain the distance, altitude, angle, etc., of the flight of the UAV.

A. Constraint on the Maximum Path

Because of fuel constraints or mission requirements, the maximum path length must be defined, and the length of a UAV path must not be longer than the maximum path length. The constraint conditions are:

(8) $$\sum_{i=1}^{n}\left|l_{i}\right|\leq L_{\max}$$

where *L_max_* means the maximum path length.

B. Constraint on the Minimum Ground Clearance

In order to avoid a collision between the UAV and the ground during the flight, this paper set the minimum ground clearance for the UAV. The height of the UAV and the flight process must be higher than or equal to the minimum ground clearance constraint, defined as

(9) $$h_{i}\geq h_{\min}$$

where *h_min_* means the minimum ground clearance.

C. Constraint on the Maximum Climb Angle

A maximum climbing or descending angle must be set during a UAV flight to ensure the safety of the UAV flight. The constraint condition of its maximum climbing angle was:

(10) $$\frac{\left|z_{i}-z_{i-1}\right|}{a_{i}}\leq \tan\theta_{\max}$$

where $\theta_{\max}$ means the maximum angle, $\left|z_{i}-z_{i-1}\right|$ means the height difference of path section *i*, and *a_i_* is the horizontal projection length of path section *i*.

## 4. Overview of HHO

The HHO algorithm uses mathematical formulas to simulate the predation strategy of Harris hawks under different mechanisms in a real environment. In the HHO algorithm, the Harris hawks represent candidate solutions, and the prey becomes gradually close to the optimal solution through continuous iterations. The HHO algorithm consists of two stages: global exploration and local exploitation.

The important condition for the accurate operation of the HHO algorithm is to maintain a proper balance between global exploration and local exploitation, and their transition is mainly achieved through the prey’s energy equation, mathematically expressed as:

(11) $$E=2E_{o}\left(1-\frac{t}{T}\right)$$

(12) $$E_{o}=2*rand-1$$

where *E* is the prey’s escape energy, *E*_0_ denotes the initial energy state of the prey, *T* means the maximum number of iterations, and *rand* represents a random number between 0 and 1. When |*E|* ≥ 1, HHO enters the global exploration stage, while when |*E|* < 1, HHO enters the local exploitation stage.

### 4.1. Global Exploration

In the global exploration stage, the Harris hawks search and monitor the space within [*lb*, *ub*] and randomly search for prey according to two strategies. In the iteration process, they update their position with a probability of *q*, as shown in the following formula.

(13) $$X_{t+1}=\{\begin{array}{c} X_{rand}-r_{1}\left|X_{rand}-2r_{2}X_{t}\right|,q\geq 0.5\\ \left(X_{prey,t}-X_{average,t}\right)-r_{3}\left(lb+r_{4}(ub-lb)\right),q<0.5 \end{array}$$

where *X_t_*_+1_ and *X_t_* mean the positions of the Harris hawks in the (*t* + 1)th and *t*th iterations, respectively. *X_prey,t_* is the position of prey in the *t*th iteration, and *r*_1_, *r*_2_, *r*_3_, *r*_4_, and *q* are random numbers between 0 and 1. *X_rand,t_* is the random position of the Harris hawks in the *t*th iteration. *X_avergae,t_* is the average position of the Harris hawks with population *N* in the *t*th iteration, and the formula is as follows:

(14) $$X_{average,t}=\frac{1}{N}\sum_{i=1}^{N}X_{i,t}$$

### 4.2. Local Exploitation

In the local exploitation phase, the Harris hawks select a siege strategy based on the range of the prey’s escape energy *E*, |*E*| ≥ 0.5 for soft siege and |*E*| < 0.5 for a hard siege. The random parameter *u* generated at initialization is used to represent the probability of the prey escaping. When *u* ≥ 0.5, the prey successfully escapes the siege. According to the range of the prey’s escape energy *E* and the probability of the prey escape *u*, the HHO algorithm can be divided into four strategies.

A. Soft siege

When |*E*| ≥ 0.5 and *u* ≥ 0.5, called the prey escape abundant energy state, the Harris hawks will prey, as energy consumption gradually affords the best position to dive and catch the prey. The position update strategy is as follows:

(15) $$X_{t+1}=\Delta X_{t}-E\left|JX_{prey,t}-X_{t}\right|$$

(16) $$\Delta X_{t}=X_{prey,t}-X_{t}$$

(17) $$J=2\left(1-r_{5}\right)$$

where Δ*X_t_* means the position difference between the Harris hawks and the prey during the iteration process, *J* is the prey’s random jump, and *r*_5_ is a random number between 0 and 1.

B. Hard siege

When |*E|* < 0.5 and *u* ≥ 0.5, called the strength to run out of the prey state, the escape energy *E* is extremely low. At this point, the Harris hawks will quickly surprise their prey, and the position update strategy is as follows:

(18) $$X_{t+1}=X_{prey,t}-E\left|\Delta X_{t}\right|$$

C. Soft siege with progressive rapid dives

When |*E|* ≥ 0.5 and *u* < 0.5, called the prey escape abundant energy state, the Harris hawks pounce before establishing a soft siege. In this situation, the Levy function (*LF*) [36] is integrated into HHO to simulate the prey’s jumping and escape action with the updating position strategy being:

(19) $$X_{t+1}=\{\begin{array}{c} Y:X_{prey,t}-E\left|JX_{prey,t}-X_{t}\right|,if_{}F(Y)<F(X_{t})\\ Z:Y+S\times LF(D),if_{}F(Z)<F(X_{t}) \end{array}$$

(20) $$LF(x)=0.01\times \frac{u\times \sigma }{{\left|v\right|}^{\frac{1}{\beta }}}$$

(21) $$\sigma ={\left(\frac{\Gamma \left(1+\beta \right)\times \sin(\frac{\pi \beta }{2})}{\Gamma (\frac{1+\beta }{2})\times \beta \times 2^{\left(\frac{\beta -1}{2}\right)}}\right)}^{\frac{1}{\beta }}$$

where *D* means the dimension of the problem, *S* represents a 1 × *D* random vector, *u* and *v* are random values within (0, 1), and *β* is a default constant, set to 1.5.

D. Hard siege with progressive rapid dives

When |*E*| < 0.5, *u* < 0.5, called the prey escape energy shortage state, the Harris hawks first establish a hard siege to capture the prey. Their position update strategy is as follows:

(22) $$X_{t+1}=\{\begin{array}{c} Y:X_{prey,t}-E\left|JX_{prey,t}-X_{m,t}\right|,if_{}F(Y)<F(X_{t})\\ Z:Y+S\times LF(D),if_{}F(Z)<F(X_{t}) \end{array}$$

To sum up, the HHO algorithm uses the prey energy *E* and escape probability *u* to select the appropriate predation mechanism to achieve the optimal solution.

## 5. Path Planning Algorithm Based on BINN-HHO

In this research, a multi-UAV path planning algorithm that consists of a BNN and an improved HHO with a periodic energy decline regulation mechanism (BINN-HHO) is proposed to solve the multi-UAV path planning problem in a 3D space. When the UAV sensor does not detect a dynamic obstacle, the improved HHO is adopted for multi-UAV path planning. When the sensor detects a dynamic obstacle during flight, it employs the improved BINN method to replan the local path. When the dynamic obstacles in the sensor detection area disappear, the UAVs complete the local path planning and return to the predetermined trajectory of the global path planning.

### 5.1. Global Path Planning Based on HHO with Energy Cycle Decline Mechanism

In the HHO algorithm, the size of the prey energy *E* reflects the search and capture ability of the optimal solution of the Harris hawks problem. The larger the *E*, the easier the HHO algorithm conducts global exploration. Otherwise, it is easier to perform local mining. However, in the traditional HHO algorithm, *E* decreases linearly from a maximum to a minimum in a single period. Therefore, the Harris hawk require several rounds of synergy to round up and eventually capture the prey.

Thus, for the rounds between the mathematical Harris hawks and the prey “stalking–escape” phenomenon, this research adjusted the mechanism and energy cyclical decline so that the prey energy *E* depicted the multiplicity of the game. The rounds of the Harris hawks’ stalking and the final capture of the prey were represented by the number of graded energy cycles *E*. When *E* periodically approached zero, the Harris hawks approached and captured the prey according to a probability, thus optimizing the multiple rounds of “global + local” search. In this paper, the cosine function was used to describe the periodic recursion of the prey energy *E* under this mechanism, defined as follows.

(23) $$E=2E_{0}(1-\frac{t}{T})\cdot \cos((2k+\frac{1}{2})\pi \frac{t}{T})$$

where *k* = 0, 1, … is the decreasing cycle number of the prey energy *E*. Given the excellent performance of the hHO-PD6 algorithm [34], we set *k* = 6 for the subsequent BINN-HHO experiments.

### 5.2. Local Path Replanning Based on Improved Bioinspired Neural Network

A BINN is derived from the circuit model of the nerve membrane and the action potential transfer formula proposed by Hodgkin et al. [37]. In 1988, Grossberg applied this method for motion control and path planning [38]. At present, more and more scholars apply this method in the field of UAV path planning. Compared with other algorithms, the BINN algorithm has the advantages of a high obstacle avoidance flexibility and a fast calculation speed for path planning in a complex environment. However, sometimes there are disadvantages such as a long planning path and a low security [38].

This paper improves the BINN algorithm by enhancing the activity of the target neurons on the predetermined global planning path and adjusting the network model to avoid falling into a local optimum solution, thus improving the algorithm’s efficiency. The UAV initiate local path replanning when the sensor detects a dynamic obstacle ahead. The UAV starts from the current position, and the current flight direction is used as the target area for path replanning. When no dynamic obstacle ahead is detected by the sensors, local path planning ends, and the UAV returns to the predetermined path.

The 3D task model of local path planning is shown in Figure 2. In this model, each grid cell represents a neuron, and each neuron’s information is updated every second. Every second, the activity information of the neurons is drawn according to the environmental information detected by the sensors, and each neuron is connected with its neighbors to form an active transmission network. In the basic BINN model, the UAV is placed at the center of 26 neurons, and it spends one-third of its time calculating information utilizing the backward-moving neurons, resulting in a low success rate of dynamic obstacle avoidance. Since the UAV will not fly backward when flying on a predefined trajectory, the BINN model presented in this paper places the UAV at the center of the first segment to detect the two-step environment and make the information transmission of each neuron more efficient.

(24) $$\frac{dx_{i}}{dt}=-Ax_{i}+(B-x_{i})S_{i}^{e}-(D-x_{i})S_{i}^{i}$$

where *x*_i_ represents the activity value of the *i*th neuron, *A* controls the decay rate of the neurons, and *B* and *D* represent the range of the neuron activity. $S_{i}^{e}$ stands for excitatory excitement, and $S_{i}^{i}$ is the inhibiting excitement, expressed as:

(25) $$S_{i}^{e}={\left[A_{s}\right]}^{+}+\sum_{j=1}^{n}\omega {\left[x_{j}\right]}^{+}+{\left[R_{s}\right]}^{+}$$

(26) $$S_{i}^{i}=\left({\left[A_{s}\right]}^{-}+{\left[R_{s}\right]}^{-}\right)$$

where *A_s_* and *R_s_* represent external stimuli obtained by the environment, with their values obtained by Equations (2) and (3). $\omega_{ij}$ means the connection weight of the *i*th and *j*th neurons. This article used the angle between two neurons as a criterion, and the smaller the angle, the greater the weight. [*A*_s_]^+^ is the excitatory excitement having a positive value, and [*A*_s_]^−^ and [*R*_s_]^−^ take a negative value when excitement is inhibited. *n* = 17 represents the 17 alternative neurons.

The activity value of each candidate neuron was calculated using the above formula. The UAV path selection strategy was as follows:

(27) $$P_{n}⇐x_{n}=\max_{}(x_{i},i=1,2,\ldots ,k)$$

where *k* represents the number of neurons near the neurons where the UAV is currently located, which was *k* = 17 for this work. *x_n_* represents the neuron that is currently most active, and *P_n_* is its location.

When the UAV selects a path, it selects the neuron with the highest activity as the next step by comparing the activity of adjacent neurons. These steps are repeated until the sensors cannot detect dynamic obstacles, then the local path planning is completed, and the UAVs return to the predetermined global plan. Because the activity of the corresponding neurons is enhanced in the direction of the reference path, the disadvantage of the BINN of easily falling into a local optimization is avoided.

### 5.3. Main Frame of Path Planning Based on BINN-HHO

**Figure 4:**
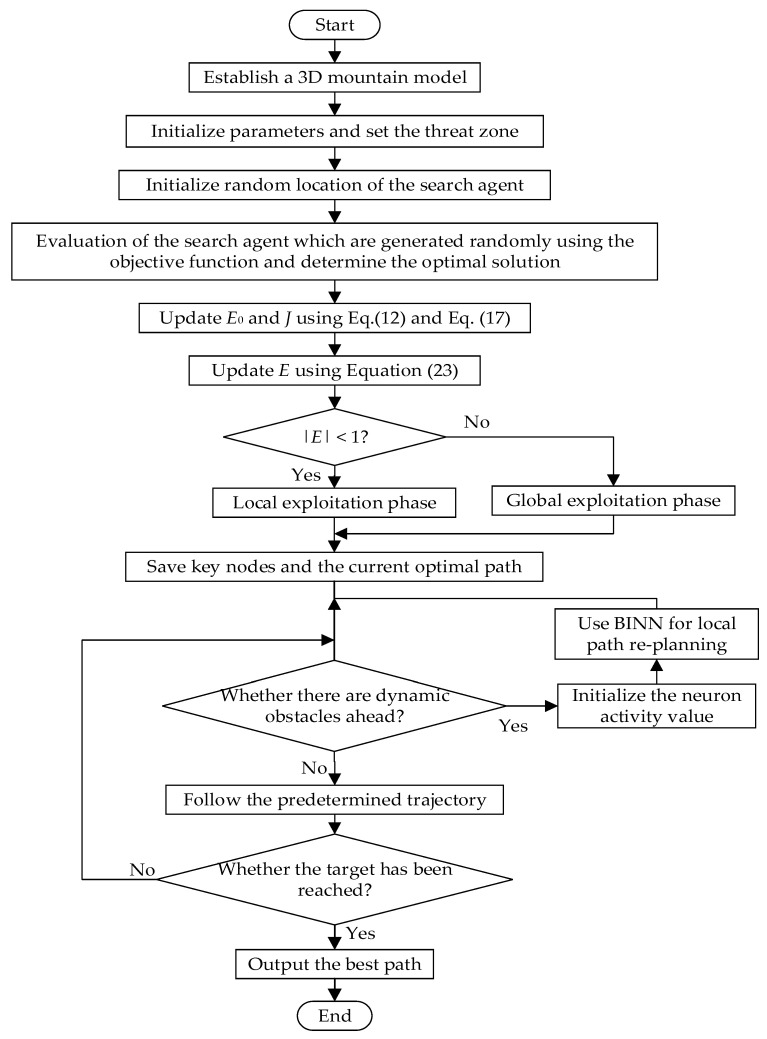
Flowchart of multi-UAV path planning based on BINN-HHO.

Based on these introductions and analyses, the pseudocode and the flowchart of the proposed BINN-HHO multi-UAV path planning are presented in Algorithm 1 and Figure 4.
**Algorithm 1** Multi-UAV Path Planning Based on BINN-HHO**Inputs**: The starting point, the ending point and threat environment information**Outputs**: Optimal path length and corresponding trajectory diagramInitialize the random population *X_i_* (*i* = 1; 2; … *N*)**While** (*t* < *T*)     Calculate the fitness value of Harris hawks;    Set the parameter *X_prey_* as the best position of the prey;    **for**(each Harris hawk *(X_i_*)) **do**    Update the initial energy *E*_0_ and jump strength *J* using Equation (12) and Equation (17);    Update *E* using Equation (23);    **If** (|*E*| ≥ 1) **then**           //   Exploration stage    Update the location vector using Equation (13);    **If** (|*E*| < 1) **then**           //   Exploitation stage    **If** (*u* ≥ 0.5 and |*E*| ≥ 0.5) **then**   //   Soft siege    Update the location vector using Equation (15);    **If** (*u* ≥ 0.5 and |*E*| < 0.5) **then**   //   Hard siege    Update the location vector using Equation (18);        **If** (*u* < 0.5 and |*E*| ≥ 0.5) **then**   //   Soft siege with progressive rapid dives    Update the location vector using Equation (19);        **If** (*u* < 0.5 and |*E*| < 0.5) **then**   //   Hard siege with progressive rapid dives    Update the location vector using Equation (22);    **end**    **end**    **end**  **end**  **for *i*** = 1: node    **if** (obstacle_flag = 1)     %If the radar detects an obstacle      Initialize the neuron activity value;      target = ***i*** + 1;       %Set the next global node to be the target point of the local programming      **while** Reach the target point do    Use Equation (2) and Equation (3) to assign value to the state information of each neuron according to the environmental information    Calculate the activity value of each alternative neuron by Equation (24)    Select the neighboring neuron with the highest activity as the next step length by Equation (27)    Update the location of neurons      **end**    **end**  **end**

The UAV path planning steps based on BINN-HHO are as follows:

Step 1: Establish a 3D mountain model, set the threat zone, and initialize the parameters.
Step 2: The global static optimal path is obtained according to the HHO algorithm using a periodic decrement mechanism.
Step 3: During the UAV flight, the sensor detects whether there is a dynamic obstacle in front of it. If there is a dynamic obstacle, the algorithm initializes the neuron activity value, and the BINN is used for local dynamic obstacle avoidance. After the dynamic obstacle disappears, the UAV returns to the predetermined orbit and follows the predetermined trajectory to reach the destination.
Step 4: Output the best path.

## 6. Experimental Results and Analysis

### 6.1. Parameter Settings

In order to analyze the BINN-HHO algorithm’s performance in multi-UAV path planning, this paper conducted an experimental simulation and analysis in two environments, including a static and a dynamic threat area. The simulation setup involved a Windows 11 64-bit operating system using an AMD Ryzen 7 5800H CPU at 3.20 GHz with 16 GB memory, equipped with a Radeon graphics card. The simulation software environment was MATLAB 2018b.

The size of the simulated mission space was 150 × 100 × 20 km and the space contained 2–3 dynamic or static threat areas. For fairness and to enhance the experiment’s objectivity, the overall scale *N* of all competitor algorithms was set to 30, and the maximum number of iterations T was set to 200. The coordinates of the start point and end point were set as (10, 50, 5.57) and (130, 10, 6.38), respectively. The standard parameters of the four algorithms were the same. The initial parameters of BINN-HHO and the information for the starting and ending points are reported in Table 1.

### 6.2. Static Environment Contrast Experiment

To verify the efficiency of the proposed BINN-HHO algorithm in path planning under a static threat environment, a comparative simulation experiment was conducted between BINN-HHO and the other three algorithms in different static environments. Table 2 shows the threat area information in the path planning.

A. Single-UAV Path Planning under a Static Environment

**Figure 5:**
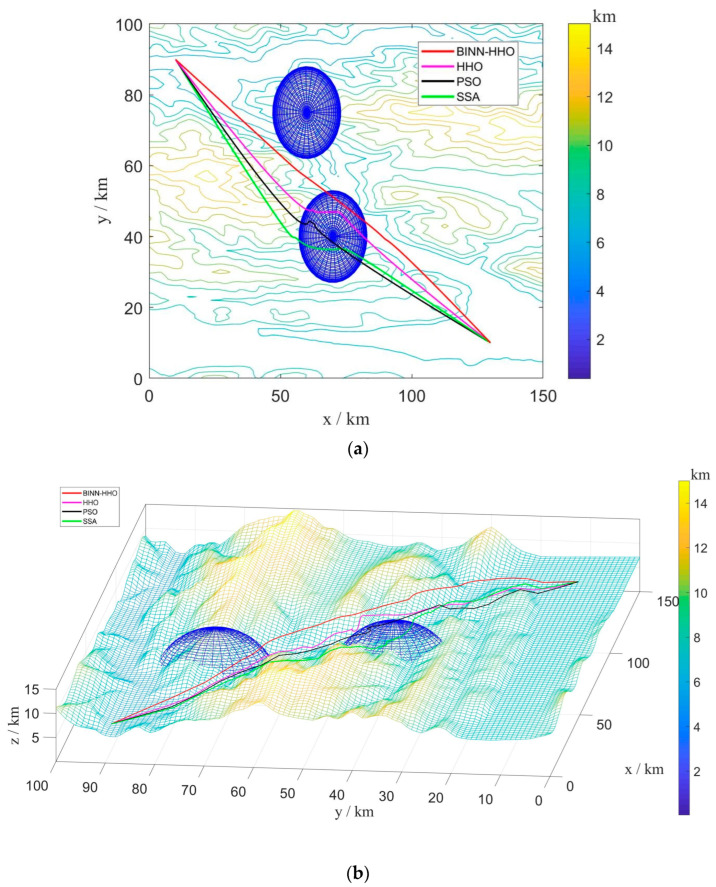
Single-UAV path planning based on BINN-HHO in a static environment: (**a**) 2D; (**b**) 3D.

**Figure 6:**
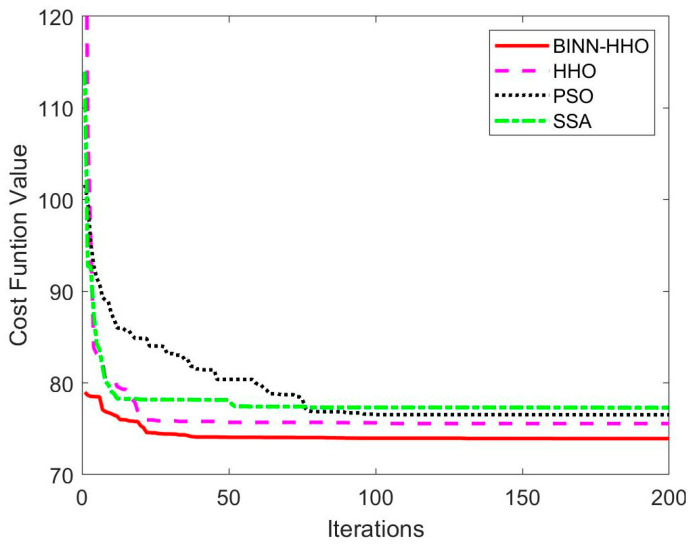
Evolution curves of cost function values in a static environment.

**Figure 7:**
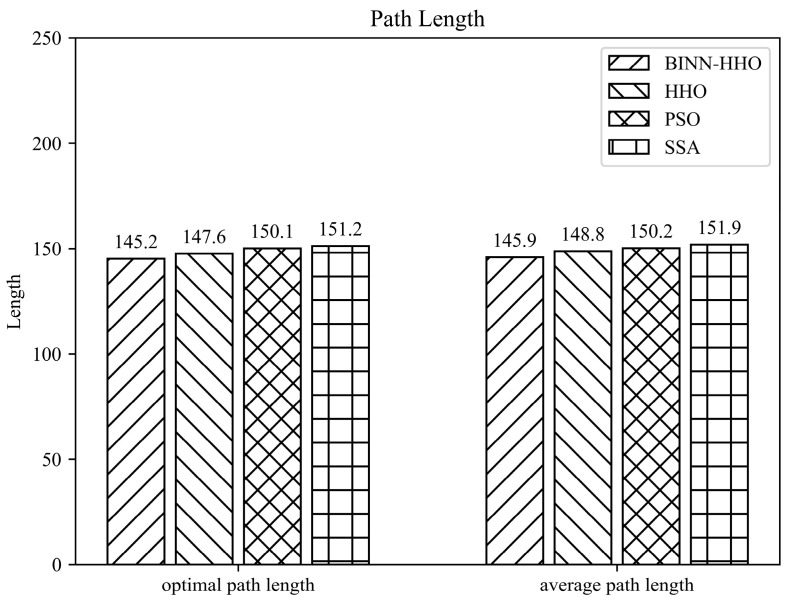
Path length of the competitor algorithms (unit: km).

The developed BINN-HHO algorithm was applied for the path planning of a single UAV under a static environment in static case I and compared with the HHO, PSO, and SSA algorithms. The final 3D simulation result is illustrated in Figure 5, and all algorithms’ substitution values and convergence speeds are compared in Figure 6. The four algorithms were tested 50 times, and their optimal and average path lengths are presented in Figure 7.

Figure 6 highlights that in the same environment, the convergence speed and optimal value of the BINN-HHO algorithm were significantly better than those of the HHO, PSO, and SSA algorithms. The cost function value of BINN-HHO was 2.2% lower than that of the HHO algorithm. Additionally, Figure 7 reveals that the average path length planned by BINN-HHO was the shortest and was 1.9% shorter than that of the HHO algorithm. The results proved that introducing the energy cycle decline mechanism could effectively and dynamically balance the global exploration and local exploitation performance of the HHO algorithm, thus improving its convergence speed, reducing the flight value, and shortening the UAV flight path length.

B. Multi-UAV Path Planning under Static Environment

**Figure 8:**
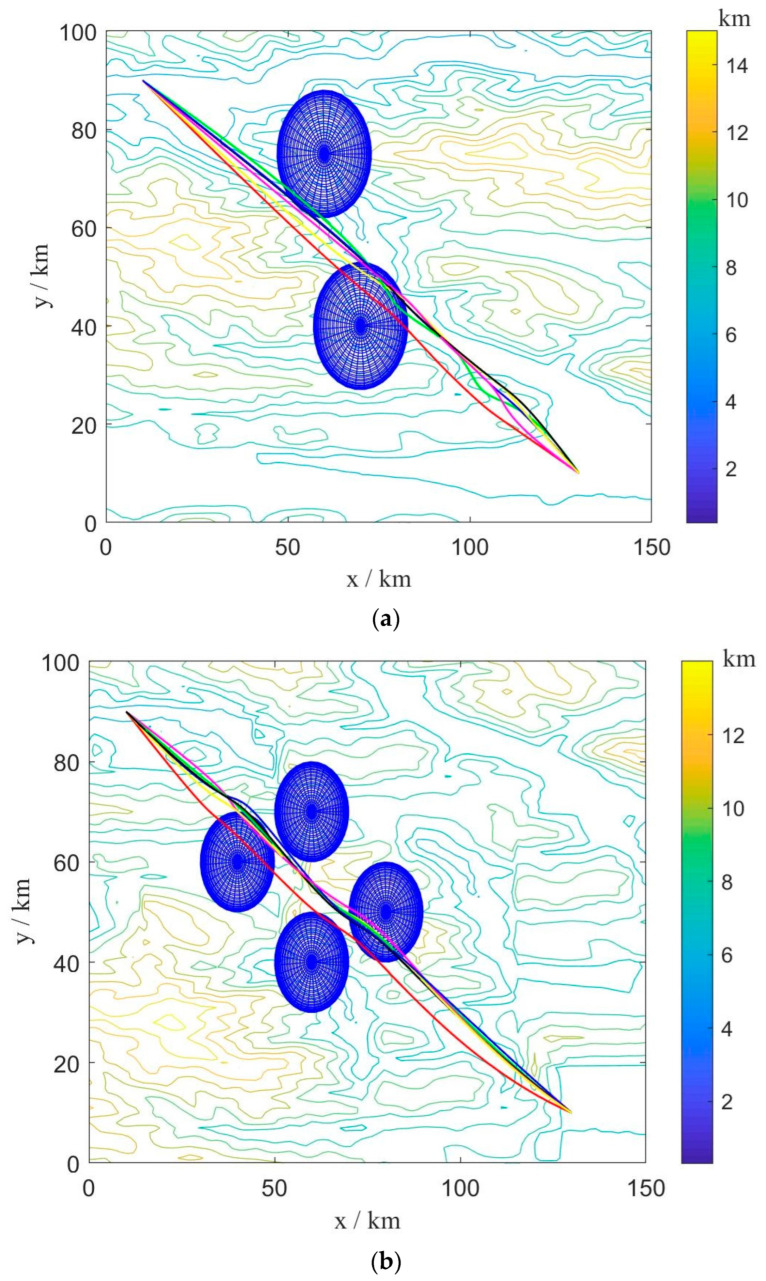
Path planning results of BINN-HHO on a 2D simulation map. (**a**) Static case I. (**b**) Static case II.

**Figure 9:**
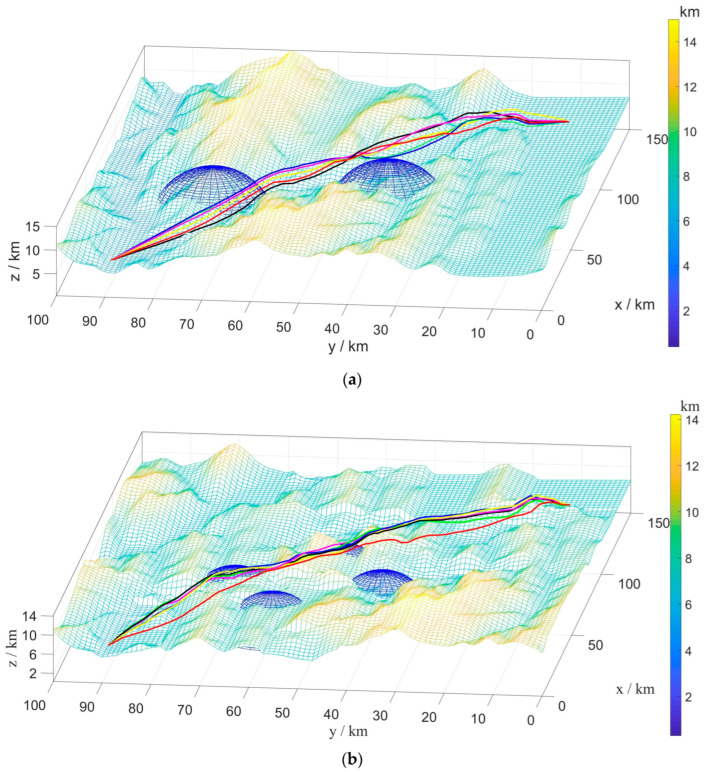
Path planning results of BINN-HHO on a 3D simulation map. (**a**) Static case I. (**b**) Static case II.

**Figure 10:**
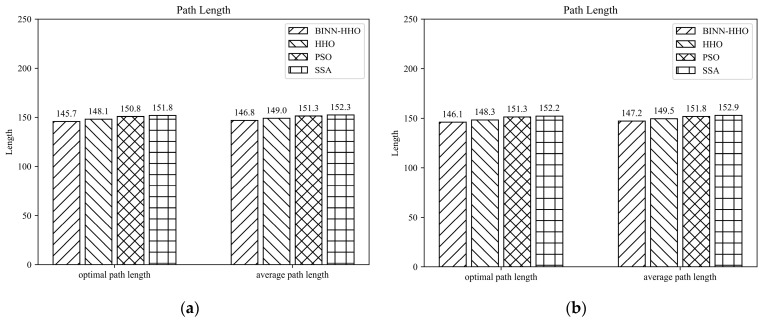
Path planning results of BINN-HHO in a static environment (unit: km). (**a**) Static case I. (**b**) Static case II.

In order to verify the effectiveness of the BINN-HHO algorithm in multi-UAV path planning with static threats, the simulation was conducted in environments with different terrain and several threats, recorded as static case I and static case II, with the corresponding environment information listed in Table 2. Similarly, the proposed BINN-HHO algorithm was compared with the HHO, PSO, and SSA algorithms, with Figure 8 and Figure 9 illustrating the simulation results, and Figure 10 comparing the path lengths of the competitor algorithms. The multi-UAV had the same start and end points, and the information is shown in Table 1 above.

Figure 10 highlights that the average length of the six UAV paths planned by the BINN-HHO algorithm was 1.5% shorter than that of the HHO algorithm in both environments. Moreover, the experimental results revealed that for single and multi-UAVs, the proposed path planning algorithm had a higher efficiency and could generate shorter planned flight paths.

### 6.3. Dynamic Environment Experiment

To prove the dynamic obstacle avoidance ability of the BINN-HHO algorithm in different dynamic environments, on the basis of the static threat area information of static case I and static case II, dynamic barriers were added, which were denoted as dynamic case I and dynamic case II, respectively. Table 3 shows the specific information on dynamic obstacles of dynamic case I and dynamic case II. The dynamic obstacles moved at a speed of 3 km/s.

A. Single-UAV Path Planning under Dynamic Environment

The proposed BINN-HHO algorithm was applied to a single UAV under a dynamic environment and compared with the HHO, PSO, and SSA algorithms. Two different environments were tested, dynamic case I and dynamic case II, and the detailed environment information can be seen in Table 3.

**Figure 11:**
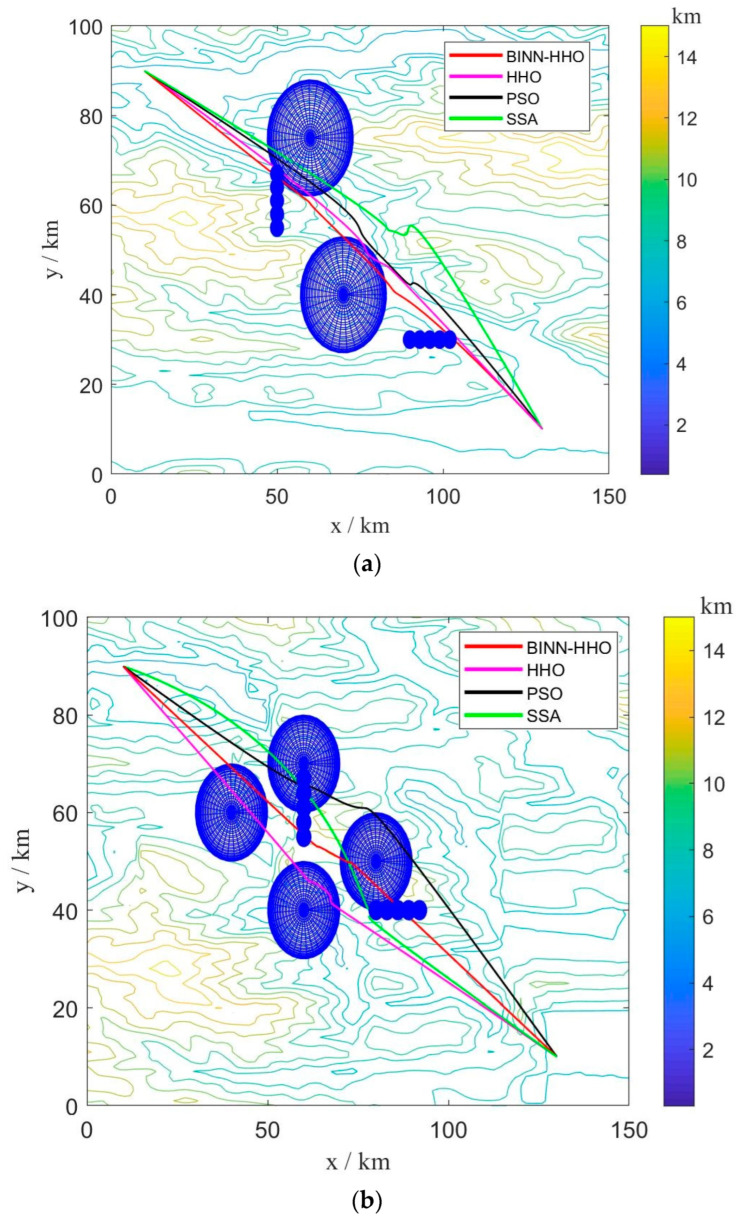
Single-UAV path planning results of BINN-HHO on a 2D simulation map. (**a**) Dynamic case I. (**b**) Dynamic case II.

**Figure 12:**
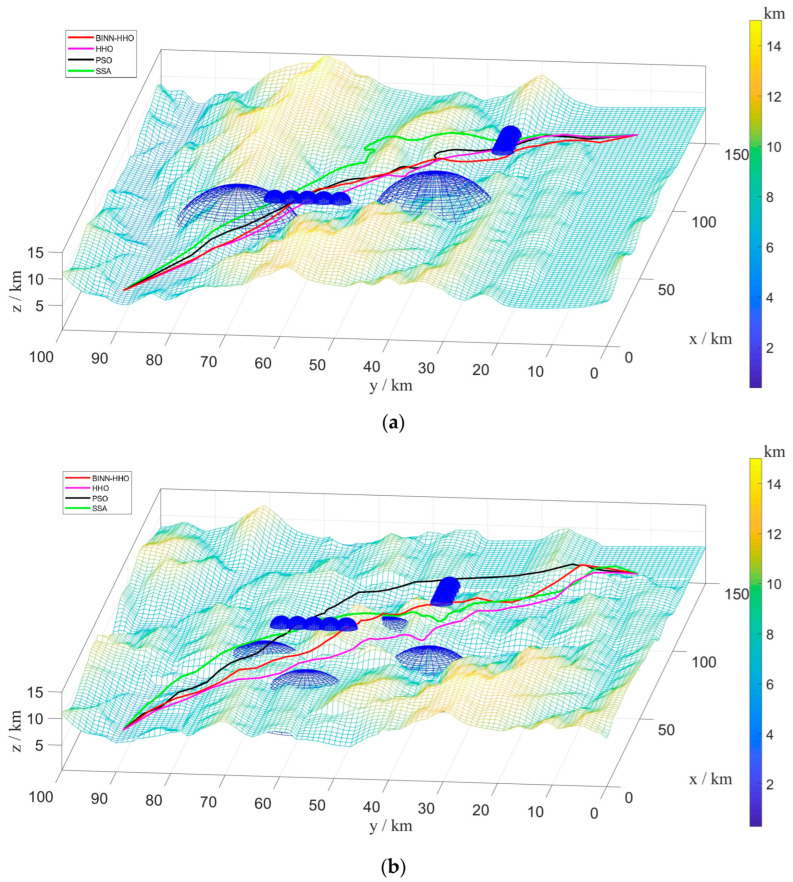
Single-UAV path planning results of BINN-HHO on a 3D simulation map. (**a**) Dynamic case I. (**b**) Dynamic case II.

**Figure 13:**
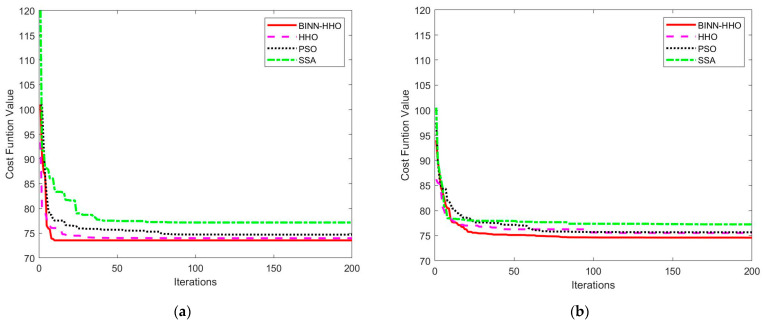
Evolution curves of cost function values in a dynamic environment. (**a**) Dynamic case I. (**b**) Dynamic case II.

**Figure 14:**
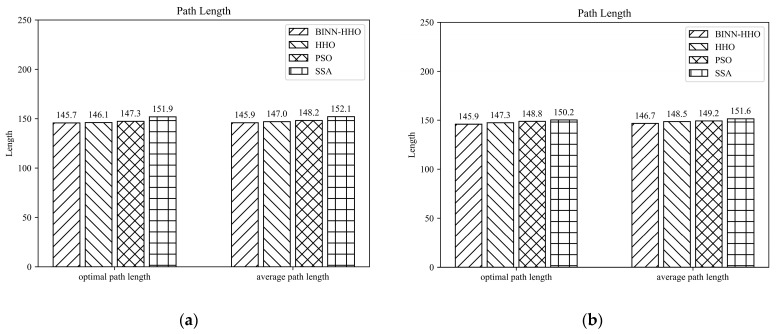
Single-UAV path planning results of BINN-HHO in a dynamic environment (unit: km). (**a**) Dynamic case I. (**b**) Dynamic case II.

The simulation results of the BINN-HHO algorithm and the other three algorithms with a single UAV in a dynamically movable obstacle environment are shown in Figure 11 and Figure 12. The four algorithms’ substitution values and convergence speeds were compared, as shown in Figure 13. The four algorithms were tested 50 times, respectively, and the statistics of their optimal and average path lengths are shown in Figure 14.

Figure 11 and Figure 12 demonstrate that when the sensor detected obstacles ahead, the UAV started from the current position, took the current flight direction as the target area and used the BINN to replan the path. When the sensors did not detect any dynamic obstacles ahead, the UAV returned to the predetermined path, and the local path planning was completed. The experimental results showed that the improved method avoided dynamic obstacles effectively and affords a collision-free flight. Furthermore, Figure 13 highlights that BINN-HHO had the best convergence value and speed, where its convergence values were reduced by 6% and 1.2% compared with those of the HHO algorithm. This demonstrated that introducing the energy cycle decline mechanism could dynamically balance the global exploration and local production performance of the HHO algorithm to obtain the optimal value quickly. 

Figure 14 reveals that the path lengths planned by the BINN-HHO algorithm in the dynamic environment of a single UAV were 0.8% and 1.2% shorter than those of the HHO algorithm.

B. Multi-UAV Path Planning under Dynamic Environment

**Figure 15:**
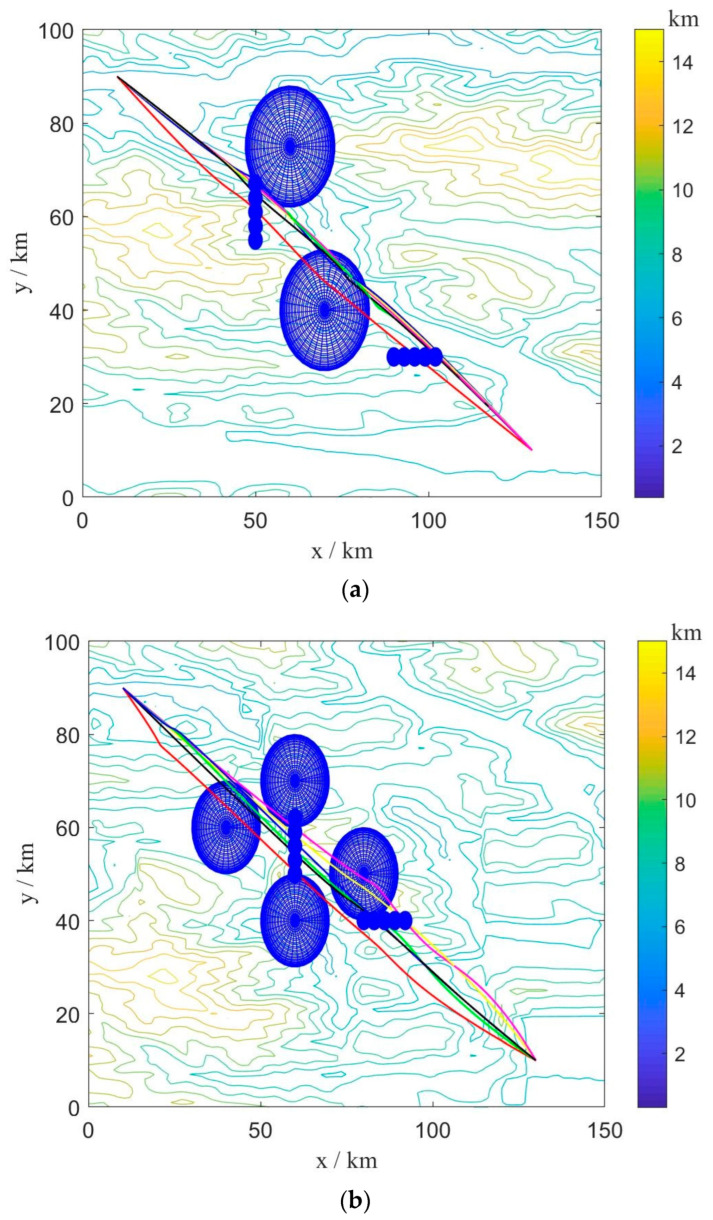
Multi-UAV path planning results of BINN-HHO on a 2D simulation map. (**a**) Dynamic case I. (**b**) Dynamic case II.

**Figure 16:**
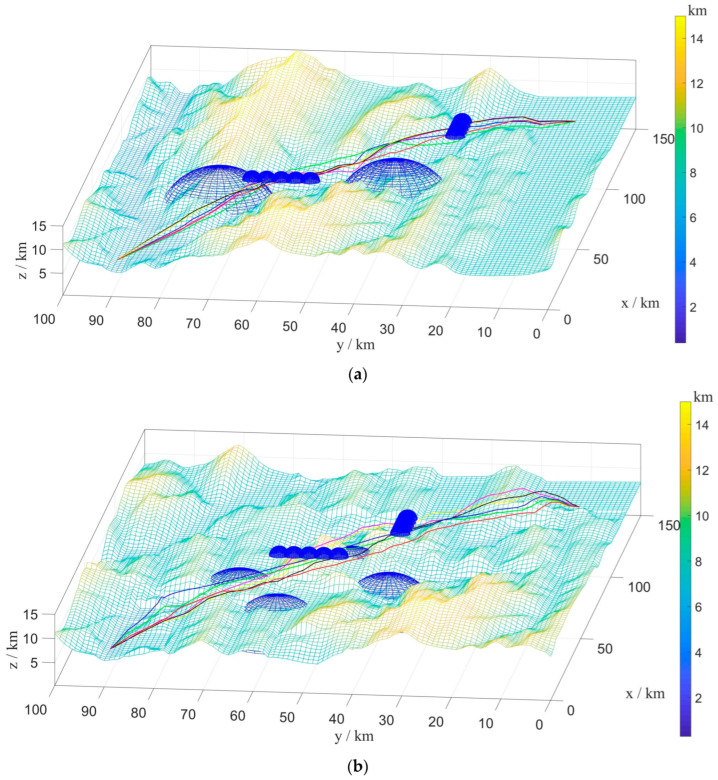
Multi-UAV path planning results of BINN-HHO on a 3D simulation map. (**a**) Dynamic case I. (**b**) Dynamic case II.

**Figure 17:**
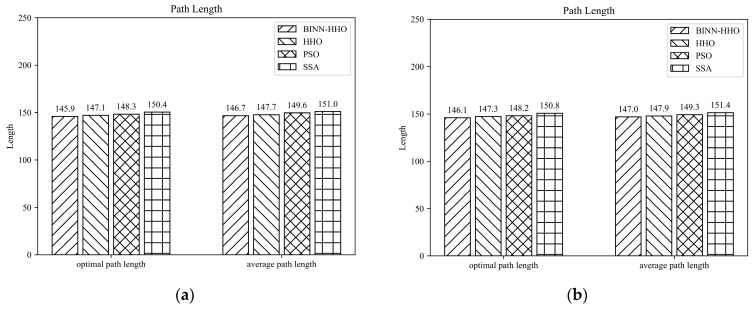
Multi-UAV path planning results of BINN-HHO in a dynamic environment (unit: km). (**a**) Dynamic case I. (**b**) Dynamic case II.

To verify the superiority of the proposed fusion algorithm in the multi-UAV dynamic obstacle avoidance environment, six UAVs were added to the above experimental environment. Similarly, multi-UAV had the same start and end points, and the information is shown in Table 1 above. The threat range information is presented in Table 3. The experimental simulation results are illustrated in Figure 15 and Figure 16, and the statistics of their optimal path length and average path length are shown in Figure 17.

The results in Figure 16 and Figure 17 infer that the six UAVs could effectively avoid dynamic obstacles flying from the front or side without colliding with mountains, threatening areas, and other UAVs.

Figure 17 demonstrates that the average flight path of six more UAVs planned by the BINN-HHO algorithm was 0.61–0.68% shorter than that of the HHO algorithm and 1.5–2.9% shorter than that of the PSO and SSA algorithms, indicating that the path length planned by BINN-HHO was shorter and more stable. The above experiments demonstrate that compared with the basic HHO, PSO, and SSA algorithms, the proposed improved algorithm is more stable in dynamic obstacle avoidance, and the planned path of BINN-HHO is shorter and better.

## 7. Conclusions

This article proposed an improved BINN-HHO algorithm for multi-UAV path planning. The 3D threat environment model was established by simulation, and the path cost function was set up, transforming the path planning problem into a multidimensional function optimization problem. On this basis, an energy cycle decline mechanism was introduced into the energy function of the HHO algorithm to improve the dynamic iterative balance between global and local search. When dynamic obstacles were detected, the BINN was activated to replan the path to assist UAVs in avoiding dynamic obstacles, allowing them to continue to fly along the predetermined path. The experimental results showed that the BINN-HHO algorithm could effectively avoid static and dynamic obstacles and obtain a safe and feasible flight path, 0.6–1.9% shorter than that of the HHO algorithm and 1.5–4.1% shorter than that of the PSO and SSA algorithms. The results highlighted that the proposed BINN-HHO algorithm had certain advantages, such as a faster convergence and a shorter flight length, in solving UAV path planning problems with static and dynamic threats. Future work will continue to focus on the obstacle avoidance problem of irregular flying obstacles in the flight process of multi-UAV. In addition, our research will always aim to find a smooth collision-free path with the shortest speed and a higher safety in other complex environments.

## Figures and Tables

**Table 1:** Initial parameters of BINN-HHO.

Parameter	Meaning	Value
ω1	Weight coefficient of path length	0.5
ω2	Weight coefficient of average flight height	0.3
ω3	Weight coefficient of comprehensive threat index	0.2
T	Maximum iteration	200
N	Population size	30
D	Problem dimension	30
Lmax	Maximum path	200
hmin	Minimum ground clearance	5
$\theta_{\max}$	Maximum climb angle	90

**Table 2:** The threat information of a static environment.

Case	Threat Area Coordinates	Radius
Static case I	(60, 75, 0)	13
	(70, 40, 0)	13
Static case II	(40, 60, 0)	10
	(60, 40, 0)	10
	(60, 70, 0)	10
	(80, 50, 0)	10

**Table 3:** The threat information of the dynamic environment.

Case	The Starting Point	Direction
Dynamic case I	(50, 55, 13)	y-direction
	(90, 30, 13)	x-direction
Dynamic case II	(60, 50, 13)	y-direction
	(80, 40, 13)	x-direction

## Data Availability

Not applicable.
